# The role of health shocks after age 70 on housing and wealth profiles

**DOI:** 10.1371/journal.pone.0312349

**Published:** 2025-01-14

**Authors:** Asiye Aydilek

**Affiliations:** College of Business and Entrepreneurship, Abdullah AlSalem University, Khaldiya, Kuwait; UCL: University College London, UNITED KINGDOM OF GREAT BRITAIN AND NORTHERN IRELAND

## Abstract

We conduct a novel investigation into the effects of uncertain health shocks and medical costs on the life cycle consumption, housing, and saving decisions. Our model aids in understanding the role of health shocks and medical costs after age 70 in explaining the lack of wealth and housing decumulation during retirement. We utilize a comprehensive life-cycle model that includes housing, as well as shocks to house price, labor income, and health. Our model could be useful for policy evaluation and future studies concerning older adults. Our first contribution to the previous literature is modeling the whole adult life-cycle. This enables us to determine whether decisions in youth and middle age are influenced by anticipated health shocks in old age. Our second contribution is modeling housing explicitly with health shocks. Conclusions regarding the savings puzzle may be significantly influenced by the explicit modeling of housing. We develop a more realistic model by relaxing some of the assumptions made in previous studies. We find that health shocks motivate the household to accumulate higher wealth before retirement. Moreover, as health shocks become more severe, individuals reduce their consumption and decumulate less wealth in old age. Health shocks help explain the flat trends observed in the housing and wealth profiles of older adults. The possible health shocks after age 70 affect the decisions in young and middle ages only marginally. The wealth profile in middle and old age is affected by health shocks.

## 1 Introduction

Healthcare expenditures represent a substantial drain on financial resources, particularly for households nearing the end of life. Items such as wheelchair ramps, grab bars, and specialized diets can be costly, and these expenses are typically borne by the individual. Our primary objective is to investigate the extent to which uncertain health shocks and medical costs occurring during old age affect life-cycle consumption, housing, and wealth profiles. Specifically, we aim to understand the impact of these factors on the lack of decumulation in wealth holdings and housing during retirement. Additionally, we explore how health shocks influence household decisions made prior to retirement.

Empirical evidence shows that household holdings of housing stock increase monotonically and remain stable in old age [1]. In other words, older adults typically do not liquidate their housing equity to finance current consumption unless faced with extreme conditions, such as the death of a spouse [2]. Minimal reductions in housing equity are observed among aging families unless there is a significant change in family status [3]. There is a growing trend of older adults maintaining mortgages into retirement and not accessing home equity [4]. This trend indicates that many retirees are not utilizing a potentially valuable financial resource that could help them cover their expenses such as healthcare costs, daily living expenses, or necessary home modifications to improve their quality of life during retirement. These findings challenge the standard life-cycle theory, which predicts significant reductions in housing, wealth, and consumption for retirees [5–7].

The retirement-savings puzzle refers to the phenomenon where retirees do not significantly deplete their wealth during retirement, contrary to the predictions of standard life-cycle models. Various explanations have been proposed, including longevity risk, bequest motives, precautionary savings, medical expense risk, housing, public long-term care aversion, and public policies like Medicaid and Social Security. Some researchers argue that wealth should decline after retirement, even with a bequest motive [8–10].

In the U.S., long-term care is covered by Medicare and private plans, and by Medicaid for the poorest. However, even with these benefits, still many households have to make substantial amount of spending. Medicare provides coverage for home nursing care and skilled nursing care, but this coverage is limited. According to the Centers for Medicare & Medicaid Services (*CMS*- Medicare Coverage), Medicare covers home health care only for patients who meet certain conditions and only for a limited period. Skilled nursing care is fully covered for the first 20 days, after which there is a copayment for days 21-100. After 100 days, patients must pay the full cost. The Kaiser Family Foundation (*KFF*- Medicare Out-of-Pocket Costs) reports that despite Medicare coverage, out-of-pocket costs for older adults can be substantial, especially for services not fully covered by Medicare. Medicare Part D provides prescription drug coverage, but there are still out-of-pocket costs, including premiums, deductibles, and co-payments. The “donut hole” or coverage gap in Medicare Part D- Drug Coverage also leads to substantial out-of-pocket expenses for prescription drugs.

[11] find that even individuals with insurance face considerable uninsured financial risk, primarily through lost earnings and unreimbursed out-of-pocket costs. Centers for Medicare & Medicaid Services (*CMS*) National Health Expenditure Data indicate that out-of-pocket expenses remain significant, particularly for services not fully covered by Medicare, such as nursing home care. [12], using Cost of Care Survey, reports that a large portion of nursing home costs must be financed out-of-pocket, as these costs are not fully covered by Medicare or private insurance. Data from sources such as the Medicare Current Beneficiary Survey (*MCBS*) indicate that out-of-pocket medical expenditures rise significantly with age. For example, average annual out-of-pocket spending for Medicare beneficiaries increases from $3,000 at age 65 to over $7,000 by age 85 [13, 14].

According to Medicaid Long-Term Services and Supports, Medicaid provides coverage for long-term care for low-income individuals, but beneficiaries often must make co-payments based on their financial status. Medicaid helps cover nursing home costs, but eligibility is means-tested, and recipients often need to spend down their assets to qualify. Hence, many older adults in the U.S. still face the risk of catastrophic medical spending despite the near-universal health insurance for the population aged 65 and over.

Many older adults expect to face significant health issues as they age, often anticipating needing help with activities of daily living and incurring high medical expenses. Data from the Health and Retirement Study(2018) and National Health Interview Survey(2020) consistently show that older adults expect a decline in health as they age, with a significant percentage reporting chronic conditions such as heart disease, diabetes, and arthritis.

Moreover, Centers for Medicare & Medicaid Services (*CMS*) data reveal that older adults, particularly those over 70, face substantial healthcare costs, especially for long-term care. Approximately 70% of individuals over age 65 will need some form of long-term care in their lifetime, with many requiring extensive and costly care as they approach their late 70s and beyond (Administration for Community Living, 2020). It is indicated that average out-of-pocket medical expenses rise from $1,100 at age 75 to $9,200 at age 95, suggesting that individuals often underestimate the magnitude of health-related expenses they will face in old age [15].

[16] highlights a gap between expectations and reality, showing that while many older adults anticipate some health declines, the actual costs and care needs often exceed their expectations. This discrepancy leads to financial strain and necessitates substantial precautionary savings.

Our framework offers a realistic and comprehensive approach to understanding household financial behavior both before and after retirement in the face of health shocks and medical costs that occur after age 70. While it is important to consider health shocks at all stages of life, our focus on the older adults is due to the heightened impact and prevalence of health issues in old age. The typical older adult receives far more medical services than those of younger ages. According to National Health Expenditure (*NHEA*) data, per capita health spending for older adults is significantly higher compared to younger age groups. Data from Centers for Medicare & Medicaid Services (*CMS*) indicates that older adults use more medical services than younger populations. Medicare beneficiaries, predominantly older adults, have higher rates of hospitalizations, physician visits, and prescription drug use. The Health and Retirement Study (*HRS*) provides detailed data showing that older adults have more frequent doctor visits, higher hospitalization rates, and greater use of long-term care services compared to younger individuals.

Centers for Disease Control and Prevention (*CDC*) data on chronic diseases and health conditions by age group shows that conditions such as cardiovascular disease, cancer, and diabetes are more prevalent in older populations, leading to more frequent and severe health shocks. Data from the Health and Retirement Study (*HRS*) consistently show that the frequency and severity of health shocks increase with age. Younger individuals (those under 50) report fewer chronic conditions and severe health events compared to older adults. The National Health Interview Survey (*NHIS*) indicates that the prevalence of chronic conditions such as heart disease, diabetes, and cancer is significantly higher in older adults. World Health Organization (*WHO*) reports that non-communicable diseases, which are major contributors to health shocks, are more common in older adults. The risk of developing these diseases increases with age, making health shocks more frequent and severe in older age groups. Therefore, focusing on older adults allows us to address the most critical period for health-related financial planning. We focus specifically on health shocks after age 70 for several reasons.

Firstly, data from the Medicare Current Beneficiary Survey indicates that average out-of-pocket medical expenditure more than doubles between ages 70 and 90 [17]. Secondly, the incidence of chronic diseases and the need for long-term care significantly increase after age 70. Over 80% of individuals aged 70 and older have at least one chronic condition, such as heart disease, diabetes, or cancer (Health and Retirement Study, 2018; National Health Interview Survey, 2020). Thirdly, effective policy design requires understanding the period when health expenses are most burdensome. Medicare data indicate that 65% of older adults’ health care spending is covered by government programs, but significant out-of-pocket expenses remain, particularly for long-term care and prescription drugs after age 70 [17]. Thus, it is crucial to focus on health shocks after age 70, as this period reflects the peak of medical expenses and care needs, driving significant financial planning and policy considerations.

Our methodology involves constructing a discrete-time stochastic life-cycle model in partial equilibrium, employing a Cobb-Douglas utility function to represent preferences for consumption and housing services, accounting for health status. Health shocks are modeled as exogenous events impacting utility and consumption, while medical expenses increase with age. The model also incorporates survival probabilities based on age and health status and accounts for pre-retirement stochastic labor income, transitioning to a constant fraction post-retirement. We differentiate between housing and non-housing consumption and include the decision to be a homeowner versus a renter.

In our simulation, we model income and house price shocks using serially uncorrelated Markov processes and simulate health shocks occurring after age 70. Using our optimal decision rules derived from state-space solutions, we calculate optimal decisions by iterating the calculations across all ages for all simulation paths. State variables are updated each period according to these optimal decisions. To generate the life-cycle profiles, we average the results from all simulations.

Our first key finding is that health shocks motivate households to accumulate higher wealth before retirement. As health shocks become more detrimental, individuals tend to decumulate their housing and wealth to a lesser extent, maintaining higher wealth levels in old age. Another significant finding is that consumption expenditure before age 50 is largely unaffected by health shocks occurring after age 70. However, after age 70, individuals significantly reduce their consumption as health shocks become more severe. This reduction in consumption expenditure helps retirees preserve their wealth to cover the expected high medical expenses later in life.

The findings suggest that policies to enhance financial preparedness for health shocks should focus on raising awareness among younger households regarding future health risks. Providing incentives for early financial planning could help households better prepare for health shocks and maintain their welfare in old age. For instance, the government could implement a policy where it provides matching contributions to individuals’ health preparedness accounts. For every dollar saved by an individual for future health expenses, the government could match a certain percentage, up to a specified limit. This would incentivize individuals to start saving early and consistently for potential health shocks.

By encouraging proactive financial strategies and early savings, these policies can mitigate the impact of health-related expenses on retirees’ financial stability. For example, individuals who begin contributing to their health preparedness accounts before the age of 35 could receive additional bonuses, such as a one-time deposit or an increased matching rate. This would encourage younger individuals to prioritize saving for future health-related costs.

Another potential policy could offer tax credits for engaging in preventive health measures, such as regular health check-ups, vaccinations, and maintaining a healthy lifestyle. These measures could reduce the likelihood and severity of health shocks in the future, thereby reducing long-term medical expenses. Alongside financial incentives, the government could run educational campaigns to inform the public about the importance of preparing for future health risks. Workshops, seminars, and online resources could be provided to help individuals understand how to effectively manage their health preparedness accounts and plan for potential health shocks. By implementing these strategies, younger households can be better prepared for future health expenses, leading to improved financial stability and welfare in retirement.

## 2 Related literature

Traditional life-cycle models, such as those proposed by [5], predict that individuals will deplete their wealth after retirement to smooth consumption over their lifetime. In a world without bequest motives and with complete markets, a household should fully annuitize and consume all wealth before death [18]. In reality, [15, 19] and show that assets of the old decrease slowly if at all, and many die with significant wealth.

[20] identify both structural and behavioral barriers that restrict the utilization of various mechanisms, such as reverse mortgages, which enable seniors to access their home equity. [21] explores the relationship between home equity and long-term care insurance demand. [22] provide empirical evidence on the trends and determinants influencing the liquidation of housing assets. They find that a relatively small proportion of retirees liquidate their housing assets, opting instead to maintain homeownership well into old age. [23] investigate retirees’ intentions and behaviors regarding home equity utilization revealing that while many retirees express an intention to tap into their home equity to finance their retirement, the actual behavior often diverges from these intentions.

[24] provide a comparative analysis of home equity extraction practices among senior households across various countries which broadens the context for understanding global trends and policies related to home equity use. For instance, in countries where there is a strong cultural emphasis on leaving a bequest, such as Japan, seniors are less likely to liquidate their home equity. We integrate the concept of health shocks into the analysis of home equity extraction, which provides a deeper understanding of the interplay between health and financial security. Their empirical study lacks a formalized model. [2], using *AHEAD* data, observe that older adults do not liquidate housing equity to finance current consumption unless faced with extreme conditions like the death of a spouse. Our paper builds on this by modeling the impact of health shocks as a specific type of extreme condition.

[3] uses empirical data to explore how home equity influences retirement behavior and financial decisions among older adults. [3] does not explicitly model or include health shocks in his analysis. We expand this perspective by incorporating health shocks as significant life events that can also lead to adjustments in housing equity. [3] does not present a formal, quantitative model.

Our paper is related to a number of papers in the savings literature that consider uncertain medical expenditures. [15] focus primarily on the impact of medical expenses on savings during the retirement period while maintaining the assumption of nonnegative assets. We extend their analysis by examining how medical expenses influence not just savings but also housing decisions, showing the dual impact on both asset decumulation and housing equity. We extend their model by considering the possibility of borrowing and also modelling the whole life-cycle instead of only the retirement period.

[25] solve each household’s optimal saving decisions using a life cycle model that incorporates uncertain lifetimes, uninsurable earnings and medical expenses to examine the degree to which households are optimally preparing for retirement. Their economic environment implies a borrowing constraint in the sense that asset balances are nonnegative in every period. Plus, they do not consider housing explicitly.

[26] address whether out-of-pocket medical expenses are persistent over time. They find that 2 or 3% of older families incur medical expenses exceeding 40% of their adjusted gross incomes. Their study is purely empirical and do not include a life-cycle model. They do not explore saving and other decisions.

[27] examines the empirical characteristics of health care costs, such as their distribution over time and their correlation with other factors. While [27] is primarily an empirical study, we integrate health shocks into a comprehensive life-cycle model that includes both housing and non-housing assets, providing a more holistic view of how health care costs influence overall financial decisions, including the decision to reduce housing stocks. [27]’s emphasis is on health care costs and their implications for retirees rather than on a full life-cycle analysis.

[28] explores how various methods of financing unpredictable health expenses impact overall saving rates in the economy. [28] used simulations to show that a Medicaid program reduced precautionary saving against uncertain medical expenses. However, his analysis relies on highly simplified probability distributions for these random health expenses, which are not grounded in real-world data. Additionally, his model assumes that medical expenses occur independently over time, which is a significant simplification.

[29] shows that uncertain medical expenses and the risk of catastrophic health costs lead to substantial precautionary savings. [29] does not explicitly include housing as a major component in his analysis. While [29] focuses on precautionary savings in response to medical expense risk, we extend this by examining not only precautionary savings but also broader financial behaviors, including changes in housing equity and consumption patterns. Our model shows how anticipated health shocks lead to a more complex set of financial decisions, beyond just savings. He focuses on the retirement period exclusively while we model the pre-retirement phase of the life-cycle as well.

[30] find that precautionary savings for potential medical expenses and social insurance are key factors in explaining why retirees do not deplete their wealth as much as standard life-cycle models predict. They focus on precautionary savings without a detailed examination of how housing assets are managed in response to health risks and uncertainties. While they assume non-negative asset balances, meaning they cannot incur debt in their retirement, our model allows for borrowing against home equity.

[31] examines how retirees allocate their wealth among bonds, stocks, and housing in the context of stochastic health depreciation. Our work extends this analysis by also considering the period before retirement, allowing us to investigate how anticipated health changes in old age may influence financial decisions earlier in life. Our approach to modeling health shocks and medical expenses differs from that of [31], providing a new perspective. While [31] models health as an accumulation process, we treat health risk as exogenous. This assumption is reasonable, as older individuals have largely shaped their health and lifestyle by this stage. Unlike [31], we abstract from investment in health. Although some medical expenses are certainly avoidable through investment, many of them are not. We believe that exploring various models of health shocks is crucial for gaining a deeper understanding of these issues. Furthermore, while [31] does not offer policy recommendations, our study provides specific guidance in this area.

Late-in-life medical expenses have a profound impact on consumption decisions. [32] demonstrate that these expenses are substantial and lead to intense savings behavior among older adults. [32] focus on the empirical analysis, do not explicitly use a life-cycle model in their study, and do not provide policy recommendations. Our paper builds on this by incorporating a more detailed and realistic modeling of medical expenses within the life-cycle framework and suggesting policies.

[33] study the roles of bequest motives and aversion to public care in explaining the lack of asset run-down and underannuitization. They assume that households are not allowed to take a negative position in assets (no-borrowing constraint). Our modelling of health shocks is different. They do not include the health state directly in the utility function, but we do. [33] primarily focus on the retirement period and it does not model the whole adult life cycle. We extend their work by integrating housing decisions into the life-cycle model, relaxing the assumption of nonnegative asset balances, including the health state directly in the utility function and modelling the whole adult life-cycle.

Housing decisions also play a critical role in this context. [34] examines portfolio choices over the life cycle and finds that housing serves as both a consumption good and a financial asset, influencing household asset allocation. [34] focuses on housing as part of overall asset allocation without considering the specific impact of health shocks. [35, 36] address life-cycle decisions, but they did not include health shocks. [37–39] also explore household decisions. However, they did not consider health shocks, house price shocks, or labor income as they model the retirement period.

[40] examine the role of housing in retirees’ (dis)saving behavior. They focus on retiree households. They abstract from the decision of a homeowner to move to a different house, or the decision of a renter to buy a house. They assume that households anticipate the increase in a deterministic fashion, and do not face idiosyncratic house price shocks. They further assume that households expect house prices to grow at a constant rate. These assumptions limit its reflection of real-world housing market dynamics. In contrast, our model is more realistic and practical. We include decisions about moving for homeowners and purchasing a home for renters, and we do not restrict house prices to only increase. We also assume that house price appreciation follows an i.i.d. normal distribution and do not assume that individuals anticipate price increases. We extend their analysis by modelling the whole adult life-cycle.

Consumption and savings decisions are further influenced by health shocks and precautionary savings motives. [41] introduces the buffer-stock saving model, where individuals hold precautionary savings to buffer against income shocks and uncertainty, complementing the life-cycle hypothesis. [41] focuses on income shocks and uncertainty in the buffer-stock saving model. We extend this model by incorporating health shocks as a specific source of uncertainty and including housing explicitly.

[1] show that consumption patterns over the life cycle are influenced by precautionary savings motives, with the market value of housing services increasing until age 55, decreasing slightly, and then flattening out, reflecting a hump-shaped consumption pattern. However, they do not explicitly model health shocks or account for borrowing against home equity.

[8] finds that bequest motives are relatively small in explaining wealth accumulation but still contribute to precautionary savings behavior. However, [8] does not explicitly model health shocks, consider housing decisions, or the possibility of borrowing against home equity, but our paper does.

## 3 Contributions to literature

Our analysis is primarily based on previous research by [15, 28–30, 42] which integrate health uncertainty into life-cycle consumption models. These studies explore how unpredictable health events influence individuals’ decisions on saving and spending over their lifetime.

Unlike previous studies that often focus on post-retirement periods (e.g., [15, 27, 29, 31, 33, 40]), we model the entire adult life cycle. This approach helps to identify whether decisions made in youth and middle age are influenced by anticipated health shocks in old age, offering a comprehensive view of financial behavior throughout the lifespan.

We extend existing models by explicitly incorporating housing decisions and health shocks. Previous models often aggregated all assets or did not consider the specific role of housing (e.g., [8, 15, 25, 30, 32, 33, 41, 43]). This explicit modeling provides a clearer understanding of the interaction between health risks and housing decisions.

We adopt a more realistic approach by relaxing restrictive assumptions about non-negative asset balances from earlier studies [8, 15, 25, 30, 33] allowing for borrowing against home equity subject to a collateral constraint. This relaxation makes our model more applicable to real-world financial practices, such as home equity loans and reverse mortgages, which older adults use to manage expenses and liquidity needs. The inclusion of a collateral constraint ensures that borrowing is limited by the home’s value, aligning with lending practices. Relative to previous literature, conclusions regarding the retirement savings puzzle change if one considers housing and motives for homeownership late in life explicitly, and separately from overall net worth of retirees. We also relaxed some assumptions about house price dynamics from [40].

Our model incorporates stochastic health-related elements, such as uncertain health status and medical expenses, which are often not included in previous models (e.g., [1, 8, 41, 43]). This addition makes our model more reflective of real-world uncertainties. Furthermore, our model accounts for stochastic labor income, an aspect not considered in some earlier studies (e.g., [8, 37–39]). We also include house price shocks, which are not addressed in some prior models (e.g., [8, 30, 40, 41, 43]).

[31] employs a constant elasticity of substitution function to model utility flow over non-health consumption and health, which contrasts with our utility modeling. [31] models health as an accumulation process rather than treating health shocks as exogenous events. This might oversimplify the dynamics of health risks, particularly in how they impact financial decisions. By modeling health shocks and utility differently from [31], we contribute to the literature in a novel way. We believe that these differences will provide a deeper understanding of health-related financial behaviors.

We explore the implications of different modeling of health shocks and medical costs which is useful for the literature. For example, our modelling differs from that of [31, 33]. Our modelling of house prices differs from that of [40].

We employ a formal model in our analysis, which represents a significant contribution to the literature compared to many previous studies that are primarily empirical, such as [8, 26, 27, 32]. By utilizing a formal model, we are able to systematically analyze and quantify the effects of various factors on financial decisions throughout the entire life cycle.

We compare the life-cycle decisions of older adults in the presence and absence of health shocks. This comparative analysis helps to isolate the impact of health shocks on wealth decumulation and housing decisions, providing clear insights into the role of health risks in financial decision-making. Our findings have significant policy implications which are provided in Introduction.

Similar to several purely theoretical influential papers such as [44, 45] in economics and finance, this paper is designed to make a significant theoretical contribution to the existing body of empirical research. Our focus is essential and valuable for several reasons:

First, we offer a detailed theoretical model that integrates various factors influencing financial decisions such as medical expenses. This framework allows for a holistic understanding of the complex interactions and mechanisms at play, which empirical studies might only partially capture. Second, the theoretical model provides a foundation that can guide future empirical research. It outlines clear hypotheses and mechanisms that can be tested and validated with real-world data, thereby directing and enriching the empirical research agenda.

Third, we include stochastic elements such as uncertain health status, medical expenses, labor income shocks, and house price shocks. By incorporating these elements, the model captures the real-world uncertainties and risks faced by individuals. This complexity is often challenging to address directly in empirical studies. Fourth, the theoretical approach allows for the exploration of scenarios and sensitivities that may not be immediately observable in empirical data. This can lead to novel insights and a deeper understanding of potential outcomes under various conditions.

Fifth, while many studies provide valuable data and insights (e.g., [15, 27]), they often focus on specific aspects of financial behavior. This paper synthesizes these findings into a coherent theoretical model, offering a unified perspective that can explain a broader range of observations. Sixth, the insights from the theoretical model can inform the design of policies aimed at enhancing financial preparedness for health shocks. By understanding the underlying mechanisms, policymakers can create targeted interventions that address the root causes of financial vulnerability among older adults.

Seventh, the model’s predictive capabilities allow for the assessment of potential policy impacts before implementation. This theoretical foresight can help in evaluating the effectiveness of proposed policies and identifying potential unintended consequences. Eighth, while empirical validation is important, it is not always necessary to conduct new empirical studies if existing literature already provides substantial evidence supporting the theoretical model. Numerous empirical studies such as [15] have already established the relationships between health shocks, medical expenses and savings decisions among older adults. Large-scale longitudinal studies such as the Health and Retirement Study (*HRS*) and the Assets and Health Dynamics among the Oldest Old (*AHEAD*) survey provide extensive empirical data that align with the theoretical predictions of the model.

Finally, the model builds on well-established life cycle and precautionary savings models, which have been extensively validated in the literature. For example, the works of [30, 41] provide a strong theoretical foundation that supports the model’s assumptions and predictions. The model’s predictions about the role of health shocks, precautionary savings, and the use of housing equity are consistent with findings from previous studies, indicating that the theoretical framework is well-grounded in existing research.

## 4 Model

We employ a basic life-cycle model that incorporates uncertain lifespans, uninsurable earnings, uninsurable medical expenses, and borrowing constraints. The individual faces several exogenous risks, such as survival and medical cost risks. Our model is set in partial equilibrium, assuming that exogenous variables follow finite-state Markov chains.

### 4.1 Preferences

We model preferences using a Cobb-Douglas utility function. While some studies, such as [37–38, 46] model household decisions using recursive preferences, several authors, including [47–49], employ the Cobb-Douglas utility function. An individual derives utility from consuming numeraire good consumption (*C*_*t*_) and housing services (*H*_*t*_). The individual also receives utility *B*(*W*_*t*_;*LB*) from bequeathing wealth *W*_*t*_.

Various objectives have been used for optimization problems (see [50–53]. In our model, the person decides *C*_*t*_, *H*_*t*_ and *M*_*t*_ with the objective of maximizing its lifetime utility. We use the within period utility function (*u*(*C*_*t*_, *H*_*t*_, *m*)) as below:

$$\begin{array}{c} u\left(C_{t},H_{t},m\right)=\frac{1}{1-\gamma }\delta \left(m\right){\left[C_{t}^{1-\alpha }H_{t}^{\alpha }\right]}^{\left(1-\gamma \right)} \end{array}$$ (1)

where the parameter *α* measures the share of housing services in the composite consumption ($C_{t}^{1-\alpha }H_{t}^{\alpha }$). The parameter *γ* measures the curvature of the utility function concerning the composite good. This functional form is widely used in the literature because its positive third derivative suggests that rational agents, when faced with uncertainty, will exhibit precautionary behavior. The parameter *δ*(*m*) determines how a person’s utility depends on health status. The person is assumed to be in good health if *m* = 2 and in bad health if *m* = 1. We define *δ*(*m*) by the following way:

$$\begin{array}{c} \delta (m)=1+\delta *(2-m) \end{array}$$ (2)

When *δ* = 0, health status does not affect utility. A negative value of *δ* implies a lower marginal utility of composite consumption when the person is in poor health. We obtain results for several negative values of *δ*. We follow [28] by modeling the effect of health status on the utility of consumption as a multiplicative constant.

For ages less than 70, the individual is alive with probability *F*(*t*) and derives utility from the consumption of numeraire and housing goods. The individual dies at period *t* with probability *F*(*t* − 1) − *F*(*t*) and derives utility from bequeathing wealth *W*_*t*_ to their offspring.

$$\begin{array}{c} F\left(t\right)=\prod_{j=0}^{t}\lambda_{j} \end{array}$$ (3)

where λ_*j*_ is the probability that household is alive at time *j* conditional on being alive at time *j* − 1. We assume that λ_0_ = 1, which implies that the person lives for at least one period. *B*(.) is the bequest function, *W*_*t*_ is the bequeathed wealth, and *LB* controls the strength of bequest motives.

An individual aged 70 or older faces exogenous uncertainties related to health status, survival, and medical expenses.

### 4.2 Health status uncertainty

We assume that the transition probability (*π*_*k*,*j*, *g*, *t*_) for health status depends on sex (*g*), current health (*k*), and age (*t*). This probability is defined as:

$$\pi_{k,j,g,t}=\text{Pr}(m_{t+1}=j|m_{t}=k,g,t),$$

where *j* denotes the new status of health.

### 4.3 Medical expense uncertainty

Health costs or medical expenses, *hc*_*t*_, are defined as out-of-pocket costs. *lnhc*_*t*_ = *hc*(*g*, *m*, *t*) + *σ*(*g*, *m*, *t*)*φ*_*t*_, where *φ*_*t*_ denotes the idiosyncratic component. Similar to [26, 27], we assume that *φ*_*t*_ has the following form:

$$\begin{array}{ll} \varphi_{t}=\zeta_{t}+\xi_{t}, & \xi_{t}∼N(0,\sigma_{\xi }^{2})\\ \zeta_{t}=\rho_{hc}\zeta_{t-1}+\epsilon_{t}, & \end{array}$$

*ϵ*_*t*_ and *ξ*_*t*_ are serially and mutually independent.

While *ξ*_*t*_ stands for the transitory component of health cost uncertainty, *ζ*_*t*_ denotes the persistent component with the autocorrelation *ρ*_*hc*_. We use quadrature methods of [54] to discretize *ϵ*_*t*_ and *ξ*_*t*_.

### 4.4 Survival uncertainty

Survival rate, *s*_*t*+1_, denotes the probability that an individual is alive at period *t* + 1 conditional on being alive at period *t* and their previous health status *m*_*t*_.

$$s_{t+1}=s(m_{t},t+1).$$

For individuals aged 70 and above, the probability that they are alive at period *t* is denoted by *G*(*t*).

$$\begin{array}{c} G\left(t\right)=F\left(69\right)\prod_{j=70}^{t}s_{j} \end{array}$$ (4)

The timing is as follows: At the beginning of each period, the individual’s health status and medical expenses are realized. Afterwards, the individual consumes and saves. Finally, the survival shock occurs. Individuals who die leave bequest.

We provide the life time utility (*U*) below:

$$\begin{array}{c} U=E\{\sum_{t=0}^{69}\beta^{t}\left(F\left(t\right)\frac{1}{1-\gamma }{\left[C_{t}^{1-\alpha }H_{t}^{\alpha }\right]}^{\left(1-\gamma \right)}+(F(t-1)-F(t))B\left(W_{t};LB\right)\right)\}\\ +E\{\sum_{t=70}^{T}\beta^{t}\left(G\left(t\right)\frac{\delta (m)}{1-\gamma }{\left[C_{t}^{1-\alpha }H_{t}^{\alpha }\right]}^{\left(1-\gamma \right)}+\left(G\left(t-1\right)-G\left(t\right)\right)B\left(W_{t};LB\right)\right)\} \end{array}$$

where *β* is the subjective discount factor and *E* denotes the mathematical expectations operator conditional on information available at time *t*. *U* is a period utility index assumed to be strictly increasing in its first argument, strictly decreasing in its second argument, twice continuously differentiable, and strictly concave. *F*(*t*) denotes the probability of being alive at time *t* before age 70 and *G*(*t*) denotes the probability of being alive at time *t* for ages 70 and above.

### 4.5 Labor income

The household receives stochastic labor income (*Y*_*t*_), which is the product of permanent income ($P_{t}^{y}$), transitory shocks (*ε*_*t*_), and the deterministic age dependent component (*e*^*f*(*t*, *Z*_*t*_)^).

$$\begin{array}{c} Y_{t}=P_{t}^{y}e^{f(t,Z_{t})}\varepsilon_{t} \end{array}$$ (5)

$$\begin{array}{c} P_{t}^{y}=P_{t-1}^{y}v_{t} \end{array}$$ (6)

Permanent income ($P_{t}^{y}$) is the product of the previous period’s permanent income ($P_{t-1}^{y}$) and *the* shock (*v*_*t*_) to permanent income of that period.

We assume that log(*ε*_*t*_) and log(*v*_*t*_) are independent, identically, and normally distributed with means such that their exponents have means of one.

During the retirement years, we assume that labor income (*Y*_*t*_) becomes constant and equal to a fraction *ψ* (replacement ratio) of the income without the transitory component just prior to retirement.

$$\begin{array}{c} Y_{t}=\psi P_{65}^{y}e^{f(65,Z_{65})}\;for\;all\;\;t>65. \end{array}$$ (7)

The generated income profile mimics those described by [30, 43].

### 4.6 Housing contract

A person can consume housing services either through renting or owning a house. $D_{t}^{o}$ is an indicator variable: if the person is a renter, $D_{t}^{o}=0$; otherwise, $D_{t}^{o}=1.$ Renters pay a constant fraction (*α*_*r*_) of the market value of the house, while homeowners pay the full market value to purchase the house. Households without sufficient savings for a down payment might prefer renting, making the down payment a potential incentive for renting rather than buying. High medical expenses could also incentivize renting instead of owning.

The house price appreciation rate ($r_{t}^{h}$) follows an i.i.d normal process with mean *μ*_*rh*_ and standard deviation *σ*_*rh*_. We assume that the house price shock is exogenous and permanent, as supported by several studies, including [34, 47, 48, 55, 56].

$$\begin{array}{c} P_{t}^{h}=P_{t-1}^{h}\left(1+r_{t}^{h}\right) \end{array}$$ (8)

The variable *H*_*t*_ denotes the consumption of housing services, which reflects not only the physical size but also the quality of the house.

### 4.7 Borrowing and saving with the riskless rate

Apart from holding home equity, a household can save in liquid assets, which earns the constant real risk-free interest rate (*r*). The net savings (or borrowing, if negative) is denoted as *M*_*t*_. We assume that the household cannot borrow non-collateralized debt. Thus, *M*_*t*_ must satisfy the following constraint:

$$\begin{array}{c} M_{t}\geq -\left(1-d\right)P_{t}^{h}H_{t}D_{t}^{o} \end{array}$$ (9)

where *d* is the down payment ratio, the individual must pay at least proportion *d* of the house value to purchase the house. If the individual does not own a house, they cannot borrow any money. The net savings (*M*_*t*_) do not include home equity.

### 4.8 Budget constraints

The list of choice variables is provided in Table 1. For time *t*, *C*_*t*_ denotes consumption, $P_{t}^{h}$ stands for house price, *M*_*t*_ symbolizes net saving, *r* denotes the risk-free interest rate, $P_{t}^{y}$ represents permanent labor income, *ε*_*t*_ denotes the transitory income shock, and *α*_*r*_ indicates the rental cost as a percentage of house value. $D_{t}^{o}$ is an indicator variable, which equals 1 if the person is a homeowner at *t* and 0 if they are a renter. We abstract from housing transaction costs to better isolate the effects of health shocks in the absence of transaction costs and to simplify the analysis.

**Table 1:** The list of choice variables of the person.

*Consumption* *of* *Housing*	*H*_*t*_
*Non* − *Housing* *Consumption*	*C*_*t*_
*Net* *Saving*	*M*_*t*_
*Home* *Ownership*	$D_{t}^{o}$

The intertemporal budget constraint for a household follows:

1) For a renter at period *t* − 1 who decides to become a homeowner in the current period *t* ($D_{t-1}^{o}=0,D_{t}^{o}=1$), the intertemporal budget constraint is given by:
  $$\begin{array}{c} C_{t}+P_{t}^{h}H_{t}+M_{t}+hc_{t}=M_{t-1}\left(1+r\right)+P_{t}^{y}e^{f(t,Z_{t})}\varepsilon_{t} \end{array}$$ (10)
  The person makes payment for consumption, health costs, and the full price of housing while also saving. The person’s income is derived from previous savings and labor income. We assume that health costs are zero before age 70.
2) For a renter at period *t* − 1 who decides to continue being a renter in the current period *t* ($D_{t-1}^{o}=0,D_{t}^{o}=0$), the intertemporal budget constraint is given by:
  $$\begin{array}{c} C_{t}+\alpha_{r}P_{t}^{h}H_{t}+M_{t}+hc_{t}=M_{t-1}\left(1+r\right)+P_{t}^{y}e^{f(t,Z_{t})}\varepsilon_{t} \end{array}$$ (11)
  The individual saves and pays the rental value of housing, consumption, and health costs.
3) For a person who is a homeowner at period *t* − 1 and decides to be a renter at the current period *t* ($D_{t-1}^{o}=1,D_{t}^{o}=0$), the intertemporal budget constraint is given by:
  $$\begin{array}{c} C_{t}+\alpha_{r}P_{t}^{h}H_{t}+M_{t}+hc_{t}=M_{t-1}\left(1+r\right)+P_{t}^{h}H_{t-1}+P_{t}^{y}e^{f(t,Z_{t})}\varepsilon_{t} \end{array}$$ (12)
  Part of the individual’s income comes from selling the house they previously purchased.
4) For a homeowner at period *t* − 1 who decides to continue being a homeowner in the current period *t* ($D_{t-1}^{o}=1,D_{t}^{o}=1$), the intertemporal budget constraint is given by:
  $$\begin{array}{c} C_{t}+P_{t}^{h}H_{t}+M_{t}+hc_{t}=M_{t-1}\left(1+r\right)+P_{t}^{h}H_{t-1}+P_{t}^{y}e^{f(t,Z_{t})}\varepsilon_{t} \end{array}$$ (13)
  This constraint ensures that the household’s net savings (*M*_*t*_) account for the previous period’s savings plus returns, current income, and expenses for consumption, housing costs, and health costs.
  Combining the above four cases, we get the following equation:
  $$\begin{array}{c} C_{t}+\left[\alpha_{r}\left(1-D_{t}^{o}\right)+D_{t}^{o}\right]P_{t}^{h}H_{t}+M_{t}+hc_{t}=M_{t-1}\left(1+r\right)+D_{t-1}^{o}P_{t}^{h}H_{t-1}+P_{t}^{y}e^{f(t,Z_{t})}\varepsilon_{t} \end{array}$$ (14)
  This equation summarizes the intertemporal budget constraint for all scenarios, accounting for both renting and owning decisions, as well as transitions between these states.

## 5 Parameter calibration

We categorize parameters into two groups: those determined independently of the model using estimations provided by others which are listed in Table 2, and those calibrated to ensure that model-generated statistics align with specific targets.

**Table 2:** The values of parameters which are taken from the other studies.

*Parameter*	*Value*
*Standard* *deviation* *of* *the* *transitory* *income* *shock*	0.27
*Standard* *deviation* *of* *the* *permanent* *income* *shock*	0.1
*Retirement* *age*	65
*Curvature* *parameter*	1.5
*Down* *payment* *ratio*	0.2
*Real* *risk* *free* *interest* *rate*	0.02
*Annual* *rental* *cost*	0.075
*The* *share* *of* *housing* *services* *in* *utility*	0.2
*Mean* *of* *housing* *appreciation* *rate*	0
*The* *volatility* *of* *housing* *return*	0.1
*House* *selling* *and* *buying* *costs*	0
*Innovation* *variance* *of* *persistent* *component* *of* *medical* *risk*	0.0503
*Innovation* *variance* *of* *transitory* *component* *of* *medical* *risk*	0

We assume that individuals begin their economic life at age 20, with *t* = 1 representing age 20. We assume that individuals live until a maximum age of 90 (*t* = 71). The mandatory retirement age is 65 (*t* = 46). This is a widely accepted benchmark in economic models, as seen in studies by [15, 43]. This age is realistic and aligns with typical retirement ages in many countries, including UK and Germany, providing a robust framework for analyzing post-retirement behavior. The conditional survival rates are taken from Anderson [57]. Life tables of the U.S. National Center for Health Statistics is used in [57] for the calibration of the annual mortality rate. We assume that the household makes decisions annually.

The age-dependent deterministic labor income profile, *f*(*t*, *Z*_*t*_), is chosen to reflect the hump shape of the labor income over the life cycle. The function *f*(*t*, *Z*_*t*_), taken from [58], is additively separable in *t* and *Z*_*t*_. *Z*_*t*_ denotes personal characteristics other than age, including marital status and household composition. [58] fit a third-order polynomial to labor income using *PSID* data. The labor income in their estimation also includes unemployment compensation, welfare, and transfers. They regress the logarithm of labor income on age dummies and dummies for other characteristics.

We set the standard deviation of the transitory income shock at 0.27 and permanent income shock at 0.1. These values are consistent with findings from [58], who calibrated these parameters using Panel Study of Income Dynamics (*PSID*) data. These shocks represent short-term income variability and long-term changes in income due to career progression or economic conditions.

The utility function, Cobb-Douglas aggregator between housing services and non-housing consumption, which is of the constant relative risk aversion (*CRRA*) class, is standard in the wealth distribution literature. Several papers, such as [34, 41, 43] use a *CRRA* utility function. This utility function allows for the modeling of preferences over different types of consumption and captures the risk aversion characteristics of individuals, making it a robust tool for analyzing wealth dynamics and distribution. It is not unreasonable to assume unit elasticity between housing services and non-housing consumption based on empirical evidence (see [59]).

We set the curvature parameter at 1.5. This parameter, which measures risk aversion, is within the standard range (1 to 6) used in life-cycle models. It captures the degree of concavity in the utility function, influencing how consumption and savings respond to changes in income and wealth. [60] uses a range of values typically around 1.5 to 3, to reflect moderate to high levels of risk aversion. [41] employs a value around 2, which is consistent with moderate risk aversion and the empirical observation of precautionary savings behavior.

In the U.S. economy, a typical down payment ratio, which is fraction of the house value that should be paid at the time of the purchase, is 20% (see [1]). For this reason, we assume that agents can borrow up to 80% of the house they want to buy. This value is standard in the U.S. housing market. It affects the borrowing constraints and the decision to purchase versus renting a home. This value is also used in models by [34, 56]. Major mortgage lenders like Fannie Mae and Freddie Mac commonly recommend a 20% down payment to avoid private mortgage insurance and secure more favorable loan terms.

We set the annual rental cost at 7.5% of the market value of the rental property. This rate is based on average rental yields in the U.S. and aligns with empirical estimates in the housing literature, such as [56]. According to Federal Reserve Economic Data, rental yields have consistently ranged around this value. [61] provides estimates of rental yields based on comprehensive data analysis. According to their findings, the average rental yield in the U.S. housing market hovers around 7.5.

The house price appreciation rate, ${\tilde{r}}_{t}^{h}$, follows an i.i.d normal process with mean *μ*_*rh*_ and variance $\sigma_{rh}^{2}.$ We assume that the housing appreciation rate $r_{t}^{h}$ is serially uncorrelated and has a mean zero. The shock to prices is thus permanent and exogenous. The mean of zero implies no expected long-term growth in real house prices, consistent with [62]. They examine long-term trends in real house prices and find that, on average, there is no expected long-term growth. We assume that there is no correlation between the housing return and the labor-income growth. The assumption that there is no correlation between housing return and labor-income growth is supported by various empirical studies such as [34, 43, 55, 56]. This assumption helps to isolate the effects of individual risk factors on household financial behavior.

The volatility of housing return is set at *σ*_*rh*_ = 0.1. This value is between the aggregate index level of [62] and the upper bound of the empirical estimates of [55]. The volatility reflects the variability in house prices, capturing risks in housing investment. [63, 64] highlight significant variability in house prices, underscoring the importance of accounting for volatility. They support the use of a 0.1 volatility parameter to reflect realistic risks in housing investments. [65] provide empirical evidence on the volatility of house prices across different markets, reinforcing the appropriateness of a 0.1 volatility assumption in modeling housing price risks.

We set the real risk-free interest rate at 0.02. This value is consistent with historical averages for real interest rates and affects the growth of savings and the opportunity cost of holding assets. Similar values are used in studies by [43, 66]. Federal Reserve Economic Data (*FRED*) indicates that real interest rates for 10-year Treasury Inflation-Indexed Securities from 1990 to 2020 have averaged around 2-3% (Federal Reserve Bank of St. Louis. (2020). “10-Year Treasury Inflation-Indexed Security, Constant Maturity” Federal Reserve Economic Data (*FRED*)). Additionally, the *IMF*’s World Economic Outlook database provides historical data on real interest rates, supporting the 2-3% average (*IMF*, 2022). Textbooks on macroeconomics and financial economics, such as [67], typically present long-term real interest rates in the 2-3% range.

We set the share of housing services in utility(*α*) at 0.2. This parameter indicates the proportion of utility derived from housing services compared to non-housing consumption. It reflects empirical estimates of housing’s importance in household utility. [34] estimates that housing services account for approximately 15-25% of total household utility. [68] estimate the proportion of utility derived from housing services, finding that housing accounts for approximately 20% of household utility. [69] estimate that housing accounts for approximately 20-30% of total household utility. [55] estimate that housing constitutes approximately 20-25% of total household utility, highlighting the significant role of housing services in household welfare.

We calibrate the parameters for the subjective discount rate (*β*) and the bequest strength parameter (*LB*). The calibration targets the average wealth-labor income ratio and the wealth of individuals over 65 relative to the average wealth. Using the two most reliable sources of data on inequality: the Survey of Consumer Finances (*SCF*) and the Panel Study of Income Dynamics (*PSID*). [70] find that an over-65 cohort owns 33% more wealth than the sample average. [71] mentions that median and mean wealth levels for individuals over 65 are higher than those of the general population. According to the *SCF*, the wealth of individuals over 65 is about 1.3 times higher than the average wealth of the overall population.

[72] examine the wealth accumulation patterns of *U*.*S*. households and report an average wealth-to-labor income ratio consistent with our target of 3.11. [73] provide comprehensive data on *U*.*S*. household wealth distribution, indicating that a net worth to labor income ratio of about 3 is typical for a broad cross-section of households. Similarly, [74] finds that the net worth to labor income ratio for middle-aged households averages around 3, which aligns closely with our target of 3.11. [43] also suggest that a net worth to labor income ratio of around 3.11 is consistent with observed data.

We choose the bequest strength parameter, *LB*, to match the model-generated statistic of over 65 wealth to average wealth ratio with its data value. One might question this target given that we aim to explain the slow reduction in wealth holdings of older adults. We use the same target in the standard model with no health shocks, however, this model still generates a high rate of wealth de-accumulation for older adults. Using this target for calibration does not lead the model to produce the slow decline in wealth holdings observed among older adults. If targeting the bequest strength parameter, *LB*, were sufficient to match the profiles, we would expect to see a slow reduction in wealth in all cases. However, some cases still exhibit rapid and significant wealth reduction despite using the same targeting for *LB*.

A summary of statistics for the model and the corresponding data is reported in Table 3. The first statistic, NetWorth/LInc, represents net worth over labor income. The second statistic, Over65W/AvgW, represents the wealth of individuals over 65 relative to average wealth. Our model produce statistics that match the targeted numbers reasonably well.

**Table 3:** Statistics.

*Statistics*	Data	*model*
*NetWorth*/*LInc*	3.11	3.09
*Over* 65 *W*/*AvgW*	1.33	1.33

We take the bequest function from [47]. Since we employ the Cobb-Douglas utility, the beneficiary’s expenditure on numeraire consumption and housing service consumption occurs in a fixed proportion of $\frac{1-\alpha }{\alpha }$. We define the bequest function as follows:

$$\begin{array}{c} B\left(W_{t};LB\right)=\frac{LB^{\gamma }}{1-\gamma }{\left[{\left(1-\alpha \right)}^{\left(1-\alpha \right)}\alpha^{\alpha }\frac{W_{t}}{{\left(\alpha_{r}P_{t}^{h}\right)}^{\alpha }}\right]}^{\left(1-\gamma \right)} \end{array}$$ (15)

This equation provides a structured approach to modeling bequest behavior within the constraints of a Cobb-Douglas utility framework, and the parameters *LB* and *γ* flexibility in capturing different bequest scenarios.

[15] find that the variance of log medical expenses is 2.15 normally. [27], using panel data from the Health and Retirement Survey (*HRS*) and the Assets and Health Dynamics of the Oldest Old (*AHEAD*) survey, find that a suitably constructed lognormal distribution can match average medical expenses. The information we used for modelling and parameterizations of health shocks, medical costs, income and survival rates are kindly provided by them. *HRS* and *AHEAD* surveys contain detailed information on health care costs, health insurance, and demographics. *AHEAD* is a sample of non-institutionalized individuals aged 70 or older as of 1993. They also find that medical expenses are highly correlated over time, with 66.5 percent of the cross sectional variance of medical expenses attributed to the transitory component and 33.5 percent to the persistent component. The persistent component has an autocorrelation coefficient of 0.922, indicating that innovations to this component of medical expenses have long-lasting effects.

Medical expenses are defined as the total out-of-pocket expenditures by individuals, including insurance premiums, drug costs, and expenses for hospital care, nursing home care, doctor visits, dental visits, and outpatient care. These expenses do not include amounts covered by public or private insurance. We set the innovation variance of persistent component of medical risk at 0.0503. This value is consistent with findings of [15, 27] regarding the long-term risks associated with medical expenses. [29] suggests that the persistent component of medical expenses significantly impacts savings behavior, although the study does not provide a specific value for the innovation variance.

We set the innovation variance of the transitory component of medical expenses to zero, implying that short-term fluctuations in medical expenses do not significantly affect long-term financial planning and behavior. This assumption is supported by literature emphasizing the predominant role of the persistent component in influencing savings and consumption patterns. For instance, [15] suggest that short-term medical expense fluctuations do not require substantial adjustments in savings behavior. [27] provide empirical estimates indicating that the variance of the transitory component of medical expenses is relatively small compared to the persistent component, justifying the zero variance assumption for models focusing on long-term financial behavior. [29] also highlights the significant impact of persistent medical expenses on precautionary savings, suggesting that transitory medical expenses have minimal effect on long-term savings behavior. Additionally, [30] underscore the predominance of persistent risks over transitory shocks in influencing savings behavior.

The income profile is hump-shaped before retirement. After retirement at age 65, individuals begin receiving pension income, which is a constant fraction of their labor income at age 64. This constant fraction is known as the replacement ratio. We set the replacement ratio at 0.6. The replacement ratio varies across different education groups. [58] calibrate the replacement ratio by dividing the average labor income of retirees by the average labor income in the last working year before retirement.

Social Security Administration provides data on average replacement ratios, indicating that a typical replacement ratio for middle-income earners is around 0.6. [58] find a replacement ratio of approximately 0.68 for their sample, which is slightly higher but close to our assumption of 0.6. [75] also examine retirement income and replacement ratios, finding that typical replacement ratios range from of 0.5 to 0.7, which aligns with our assumption of 0.6. [76] discuss replacement ratios in the context of retirement planning and find that replacement ratios are 70-80% for low-income, 60-70% for middle-income and 50-60% for high-income groups.

## 6 Optimal life cycle decisions

The parameter *δ* affects the relationship between a person’s utility and their health status. When the person is in good health, *δ* does not affect their utility. However, when the person is in poor health, the marginal utility of composite consumption decreases as *δ* becomes lower. We present results for five different cases. The straight blue line represents the case without health shocks. We also provide life-cycle profiles for the following *δ* values: 0, -0.1, -0.4, -0.8.

**Fig 1:**
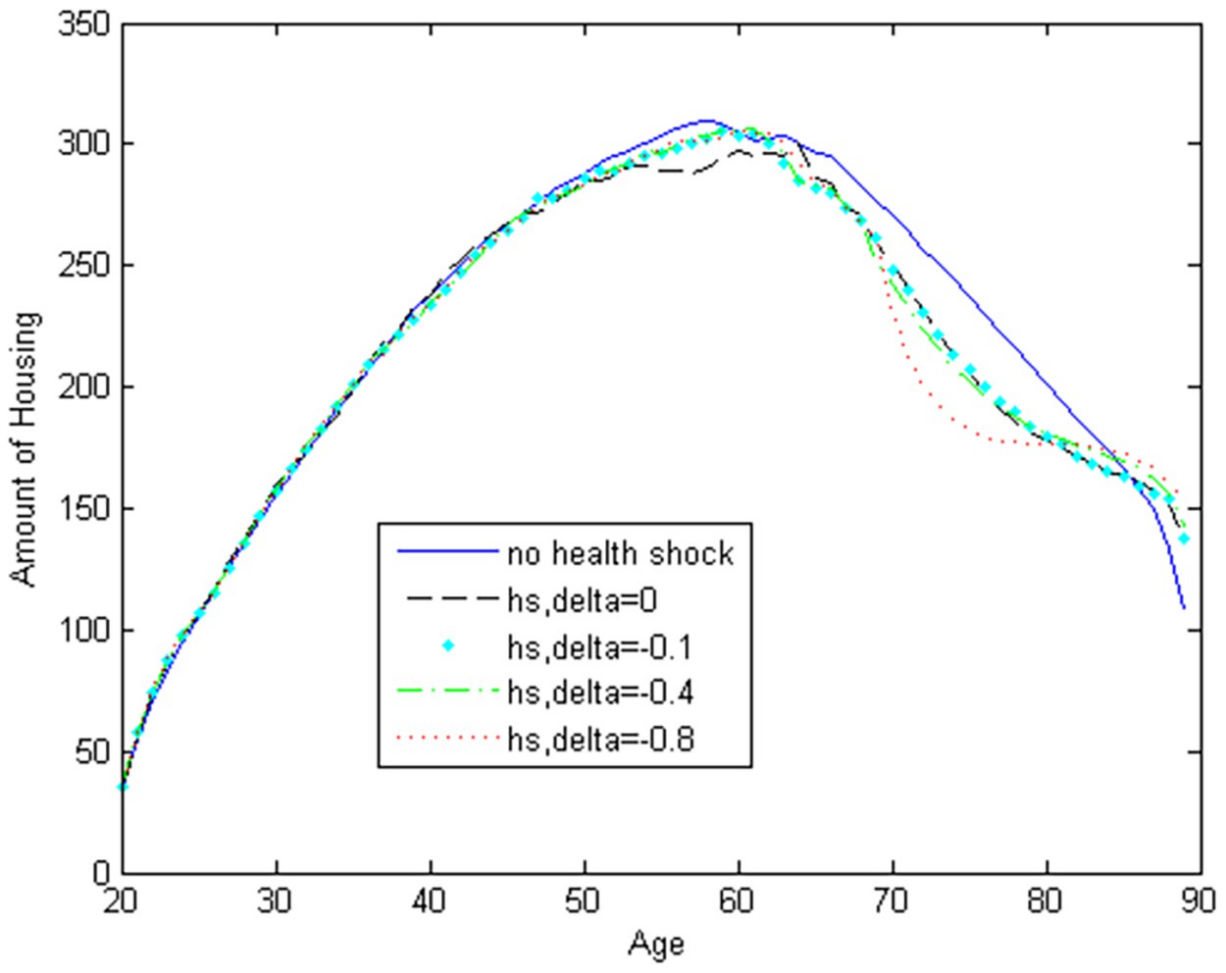
Housing with stochastic health shocks.

Fig 1 illustrates how the household’s life-cycle housing consumption varies with different values of the parameter *δ*. We observe that the decumulation in housing during retirement is highest when there are no health shocks. When *δ* = -0.8, decumulation continues until age 70, after which the housing profile becomes nearly flat. This suggests that health shocks in old age reduce the extent of housing decumulation. Therefore, health shocks help explain the flattening of the housing profile among older adults. If we extend our model to include health shocks between ages 60 and age 70, we could achieve an almost flat housing profile throughout the entire retirement period.

We also observe that the decumulation in housing after age 70 decreases as *δ* becomes lower. In other words, as health shocks become more influential in reducing the utility, individuals decumulate their housing less. The housing profile before age 50 remains unchanged with different values of *δ*. This suggests that individuals do not factor in potential health shocks after age 70 when making housing decisions before age 50. This finding is significant and should be considered in policy design. However, if health shocks prior to age 70 are also included in the model, this result might change. For example, if we model health shocks between ages 60 and 70, we might find that the housing profile before age 40 does not vary with *δ*.

To summarize, health shocks play a significant role in explaining the lack of decumulation in housing during retirement. Individuals do not factor in potential health shocks in old age while making housing decisions throughout their adult life. However, as they age and approach retirement, housing decisions are increasingly influenced by the anticipated health shocks.

**Fig 2:**
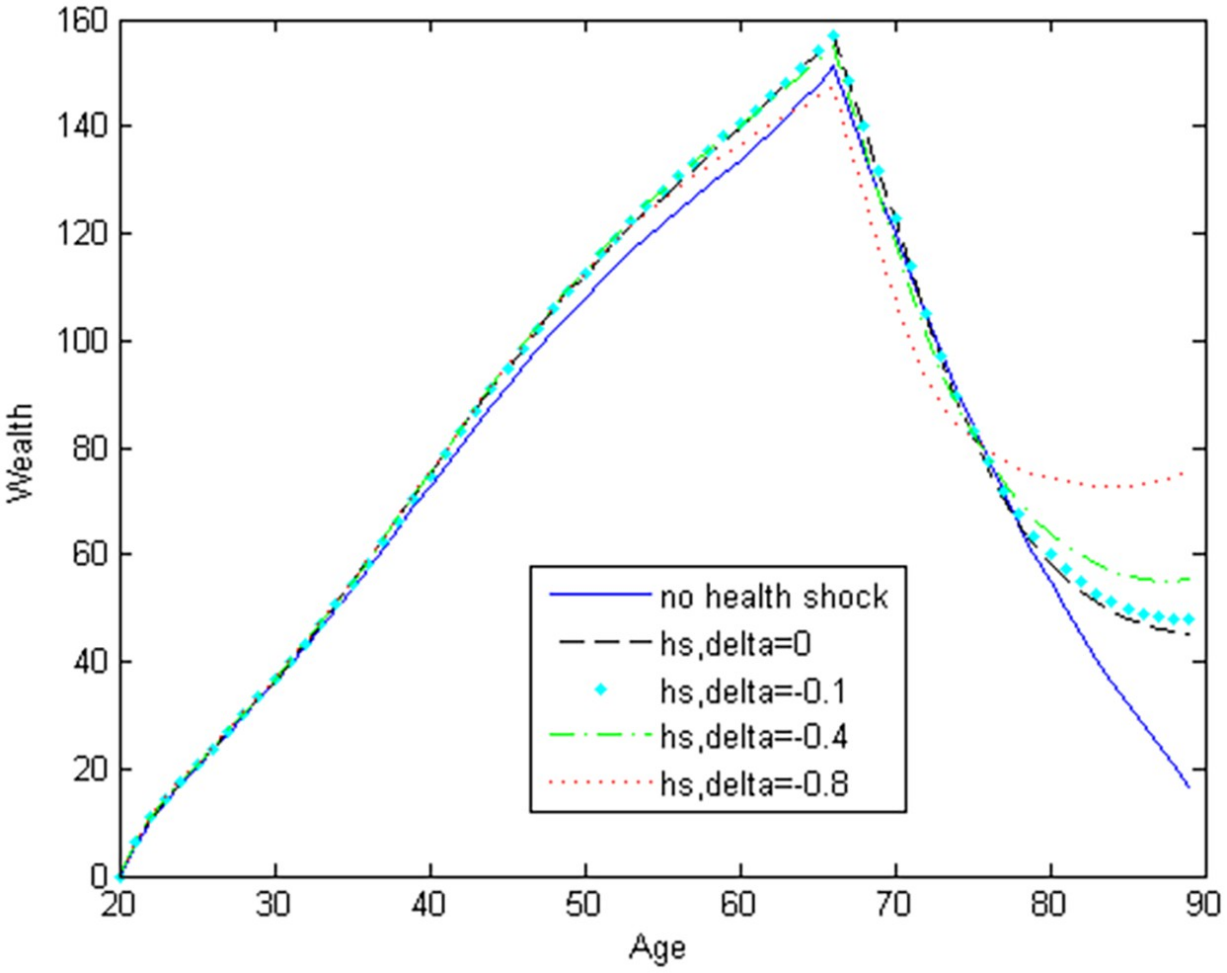
Wealth holdings with stochastic health shocks.

Fig 2 shows how the household’s life-cycle wealth profile changes with different values of the parameter *δ*. The wealth profile in young ages, which does not include housing, is almost unaffected by the *δ* value. This suggests that individuals may be too young to consider potential health shocks in old age when making decisions about their wealth accumulation. In the scenario without health shocks, individuals tend to have the least wealth before retirement. Thus, the possibility of future health shocks incentivizes households to accumulate more wealth before retirement.

We observe the greatest wealth decumulation during retirement in the scenario without health shocks. The amount of wealth decumulation during the 70s decreases as *δ* becomes lower. This suggests that health shocks help explain why older adults do not deplete their wealth significantly. In the absence of health shocks, the decumulation of wealth is considerably higher. As *δ* decreases, health shocks reduce utility more significantly, leading retirees to retain more wealth. In other words, as health shocks become more severe and detrimental (in terms of decreasing the marginal utility of composite consumption), individuals deplete their wealth more slowly.

We believe that if the model is enhanced to include health shocks before age 70, we would observe even smaller wealth decumulation. In other words, incorporating health shocks prior to age 70 might result in a flatter wealth profile for older adults. This suggests that health shocks are crucial for explaining the high levels of wealth among older adults and should be included in models that analyze decisions made by older individuals.

**Fig 3:**
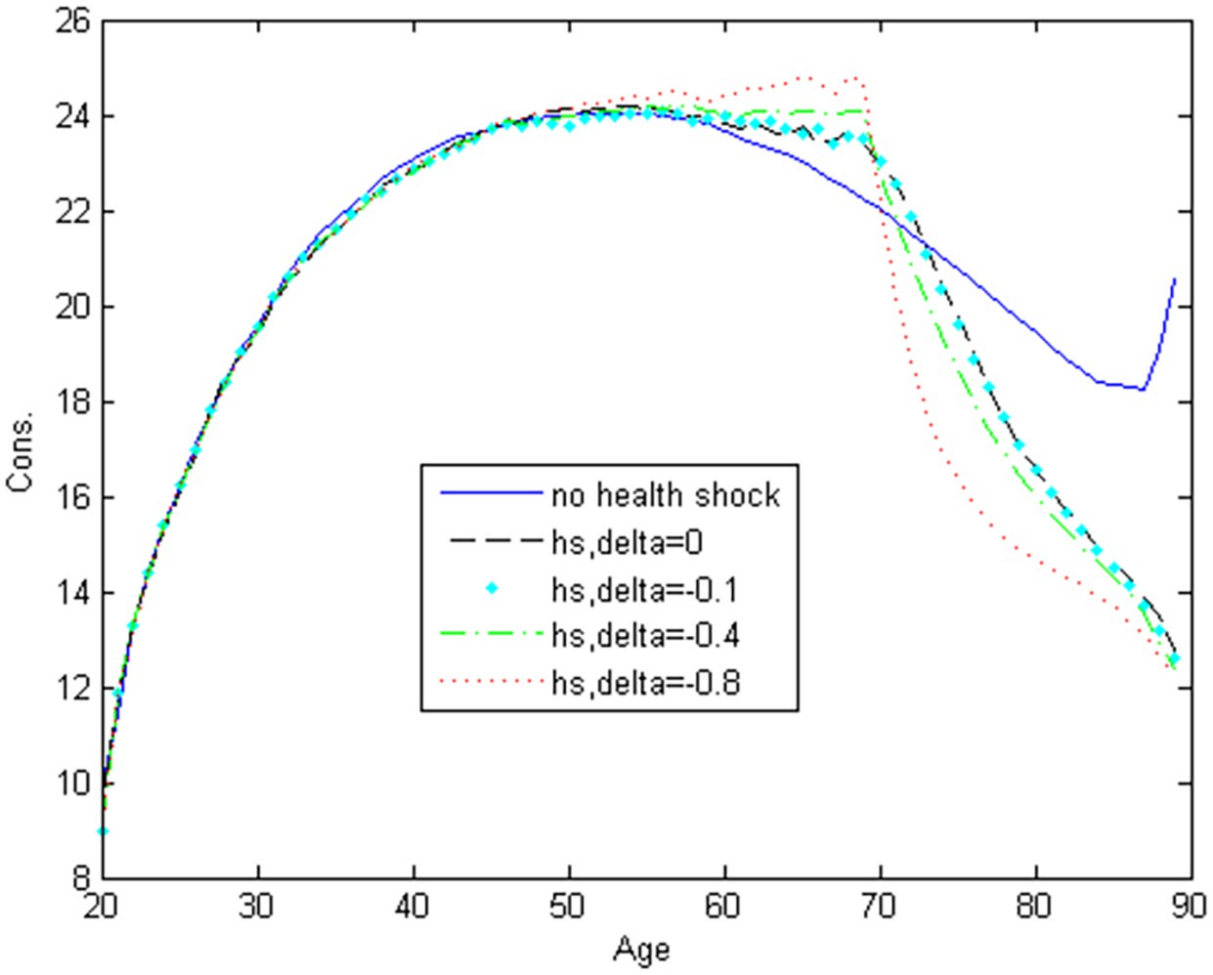
NonHousing consumption with stochastic health shocks.

Fig 3 shows how the non-housing consumption profile changes with different values of the parameter *δ*. After age 70, consumption decreases as *δ* becomes smaller. The highest consumption occurs in the scenario with no health shocks after age 70. As health shocks become more severe, retirees reduce their consumption expenditure more. This behavior allows them to retain more wealth and prepare for potential health shocks. The consumption expenditure pattern is hump-shaped in all cases, consistent with consumption profiles observed in previous literature (see, e.g., [41]. Consumption expenditure before age 50 remains relatively unchanged across all scenarios, indicating that health shocks occurring after age 70 do not significantly impact pre-50 consumption patterns. The unusual increase in consumption right before age 90 in the no-health-shocks scenario is due to the agent’s certainty of death at age 90 in the model.

## 7 Conclusion

We find that the decumulation of wealth and housing assets is less pronounced when health shocks are anticipated, supporting the idea that health risks contribute to the flat wealth and housing profiles observed among older adults. Additionally, our model shows that consumption expenditure before age 50 is almost unaffected by health shocks occurring after age 70. However, after age 70, individuals reduce their consumption significantly as health shocks become more severe. We find that retirees reduce their current consumption to cover the high out-of-pocket medical expenses they expect to incur later in life. The consumption expenditure pattern is hump-shaped in all cases, consistent with observed consumption data.

Our findings have significant policy implications. To enhance financial preparedness for health shocks, policies should focus on raising awareness among younger households about future health risks. Providing incentives for early financial planning can help households better prepare for health shocks and maintain their welfare in old age. Our study underscores the importance of integrating health risks into financial planning and retirement policies, ensuring that individuals are better equipped to handle unexpected medical expenses.

Our findings contribute to the literature by extending previous analyses and incorporating a detailed theoretical model that considers the entire adult life cycle. By explicitly modeling housing decisions and health shocks, we provide a nuanced understanding of the factors influencing financial behavior before and after retirement. Our approach offers a more realistic assessment of how anticipated health shocks affect financial behavior, providing valuable insights for policy design.

We run the model for a variety of specifications within the standard range to shed light on how our results change with different parameter values. We change the value of one parameter at a time to see the sensitivity of our results to that parameter. Most of our results remain robust across different assumed parameter values. Hence, our results are robust across different parameter values.

Future research should focus on incorporating health shocks occurring before age 70 to provide a more comprehensive view of how early-life health events influence saving and consumption decisions throughout the life cycle. Analyzing financial decisions at the couple level, including factors like the timing of each spouse’s retirement can provide a more realistic and nuanced view of household financial dynamics. Extending the model to simulate and evaluate the impact of different policy interventions such as modifications to Medicare or Medicaid could provide practical insights for policymakers.

Investigating the role of financial literacy and education in mitigating the effects of health shocks on financial behavior could identify effective strategies for improving financial outcomes. Investigating demographic differences in responses to health shocks (e.g., by gender, socioeconomic status) could reveal disparities and help tailor policy recommendations more effectively. Extending the model to include potential behavioral responses to health shocks, such as changes in preventive health measures or lifestyle adjustments, can provide a more dynamic understanding of health and financial interactions.

Like many studies in housing literature, we assume that the person retirees at some fixed predetermined age which is exogenous to our model. In real life, different people retire at different ages and some decisions may change with retirement age. Modeling the age of retirement as a decision to the person, which is not essential for our objective, could be another extension of our paper. Exploring the effects of health shocks on the use and effectiveness of various financial instruments, including Islamic finance products (see [77]), can expand the applicability of the model to diverse financial contexts. Understanding the broader financial implications of health shocks, such as their impact on shadow banking or financial markets, can provide a macroeconomic perspective on the issue.
